# Identification and characterization of smallest pore-forming protein in the cell wall of pathogenic *Corynebacterium urealyticum* DSM 7109

**DOI:** 10.1186/s12858-018-0093-9

**Published:** 2018-05-09

**Authors:** Narges Abdali, Farhan Younas, Samaneh Mafakheri, Karunakar R. Pothula, Ulrich Kleinekathöfer, Andreas Tauch, Roland Benz

**Affiliations:** 1 Department of Life Sciences and Chemistry, Jacobs-University Bremen, Campusring 1, D-28759 Bremen, Germany; 2 Department of Physics and Earth Sciences, Jacobs University Bremen, Bremen, Germany; 3 Institute for Genome Research and Systems Biology, Center for Biotechnology (CeBiTec), Bielefeld University, Bielefeld, Germany; 4 Rudolf-Virchow-Center, University of Würzburg, Würzburg, Germany; 5 Present address: Department of Chemistry and Biochemistry, University of Oklahoma, Norman, OK 73019-5251 USA

**Keywords:** Cell wall channel, Mycolic acid, Porin, *Corynebacterium urealyticum*, Lipid bilayer membrane

## Abstract

**Background:**

*Corynebacterium urealyticum*, a pathogenic, multidrug resistant member of the mycolata, is known as causative agent of urinary tract infections although it is a bacterium of the skin flora. This pathogenic bacterium shares with the mycolata the property of having an unusual cell envelope composition and architecture, typical for the genus *Corynebacterium*. The cell wall of members of the mycolata contains channel-forming proteins for the uptake of solutes.

**Results:**

In this study, we provide novel information on the identification and characterization of a pore-forming protein in the cell wall of *C. urealyticum* DSM 7109. Detergent extracts of whole *C. urealyticum* cultures formed in lipid bilayer membranes slightly cation-selective pores with a single-channel conductance of 1.75 nS in 1 M KCl. Experiments with different salts and non-electrolytes suggested that the cell wall pore of *C. urealyticum* is wide and water-filled and has a diameter of about 1.8 nm. Molecular modelling and dynamics has been performed to obtain a model of the pore. For the search of the gene coding for the cell wall pore of *C. urealyticum* we looked in the known genome of *C. urealyticum* for a similar chromosomal localization of the porin gene to known *porH* and *porA* genes of other *Corynebacterium* strains. Three genes are located between the genes coding for GroEL2 and polyphosphate kinase (PKK2). Two of the genes (*cur_1714* and *cur_1715*) were expressed in different constructs in *C. glutamicum* Δ*porA*Δ*porH* and in porin-deficient *BL21 DE3 Omp8 E. coli* strains. The results suggested that the gene *cur_1714* codes alone for the cell wall channel. The cell wall porin of *C. urealyticum* termed PorACur was purified to homogeneity using different biochemical methods and had an apparent molecular mass of about 4 kDa on tricine-containing sodium dodecyl sulfate polyacrylamide gel electrophoresis (SDS-PAGE).

**Conclusions:**

Biophysical characterization of the purified protein (PorACur) suggested indeed that *cur_1714* is the gene coding for the pore-forming protein in *C. urealyticum* because the protein formed in lipid bilayer experiments the same pores as the detergent extract of whole cells. The study is the first report of a cell wall channel in the pathogenic *C. urealyticum*.

## Background

The outer membrane of mycolata represents a permeability barrier similar to that of Gram-negative bacteria and needs specialized pore-forming proteins, called porins, to facilitate the passage of water-soluble solutes. Selective transport of molecules through the cell wall is crucial to the life-or-death of bacterial cells [1, 2]. They continuously need to exchange small molecules, nutrients and proteins with the exterior environment, while they need to keep toxic substances out [3–5]. Currently almost 100 species of the genus *Corynebacterium* are known, which are divided in three different groups: human pathogens, animal pathogens, and non-pathogens [6, 7]. Many species within the group of mycolic acid containing bacteria are important either because of their medical or biotechnological relevance. Non-pathogenic corynebacteria are used for the production of amino acids such as L-glutamate and L-lysine at industrial scale [8].

Prominent pathogens are *Corynebacterium diphtheriae* [9, 10] the etiological agent of diphtheria, *Corynebacterium jeikeium* [11], a resident of human skin and *Corynebacterium urealyticum* [12, 13]. *C. urealyticum*, formerly classified as *Corynebacterium* group D2 is one of the more common *Corynebacterium* species isolated from human clinical specimens, mainly from patients suffering from urinary tract infections [14–16]. *C. urealyticum* represents a Gram-positive, aerobic, non-spore forming, slow growing and multidrug resistant bacterium [16]. Multidrug resistance of *C. urealyticum* DSM 7109 is mediated by transposable elements, conferring resistances to macrolides, lincosamides, ketolides, aminoglycosides, chloramphenicol, and tetracycline [13, 17, 18]. *C. urealyticum* strains are opportunistic human pathogens, commonly found on the skin of hospitalized persons and they eventually might lead to urinary tract infection [19]. This organism has also been isolated from the skin of 25–37% healthy elderly individuals, mainly females [16, 19].

In general, *C. urealyticum* is highly resistant to β-lactams and aminoglycosides, and occasionally susceptible to fluoroquinolones, macrolides, ketolides, rifampicin, and tetracyclines [16, 18, 20, 21]. *C. urealyticum* has also been detected as a rare pathogen in the urinary tract of small animals, such as cats and dogs [22, 23]. The bacterium possesses a strong urease activity and this activity leads to the formation of struvite (ammonium magnesium phosphate) stones by increasing the pH and ammonium precipitation [17, 24]. The efficiency of its treatment is often affected by multiple resistance of *C. urealyticum* to a broad range of antibiotics [18, 25].

As mentioned above communication between a bacterium and its environment is essential for the survival of bacterial cells [3, 5, 26]. Many of these processes involve channels in the cell wall. *C. urealyticum* was also thought to have cell wall channels for the transport of hydrophilic solutes across the cell wall. In the closely related *Corynebacterium* species *C. glutamicum* and *C. efficiens*, *porA* and *porH* genes encode for PorA and PorH proteins, which assemble for large, water-filled cell wall pores [27–30]. We could also previously demonstrate that a channel-forming protein, named PorACj, was identified in the known genome of *C. jeikeium* by the similar chromosomal localization of its gene to the known *porH* and *porA* genes of other *Corynebacterium* strains [31]. However, in contrast to certain *Corynebacterium* species, where two polypeptides PorA and PorH are needed to form a functional cell wall pore, the pore in the cell wall of *C. jekeium* is formed by a single polypeptide PorACj [31].

Protein homology search allows the study of the evolutionary relationship between proteins, since homologous proteins share likely the same function. Analysis of sequence similarity of related proteins can be useful for functional annotation of proteins. For the search for porins in *C. urealyticum* that are homologous to the known porins from *Corynebacterium* species we used a similar approach here by using the NCBI BLAST-translation tool search [32, 33].

This alignment allowed an interesting comparison of the localization of genes coding for PorA and PorH within the genomes. Two genes coding for porins are present in the genomes of all these *Corynebacterium* species with the exception of *C. jeikeium*, where the genome contains only one gene*.* In all other cases, the genes are located in tandem between the genes coding for the chaperone GroEL2 and the polyphosphate kinase PKK2 [30]. So far, we demonstrated that PorACj is the smallest polypeptide forming well defined and stable channels [31]. The complete genome sequence of *C. urealyticum* DSM 7109 is known [13]. The search for the gene coding for the cell wall channel in case of *C. urealyticum* DSM 7109 provided a very interesting result because there exist three open reading frames (ORFs) located between the genes coding for GroEL2 (*cur_1716*) and PKK2 (*cur_1712*). It is not possible to clearly identify which of the interjacent genes *(cur_1713, cur_1714 or cur_1715)* could code for channel-forming proteins. The additional question was if one of the three ORFs was coding for a cell wall channel alone or together with one (or two) of the other two genes. These questions were also addressed in this work. The results suggested that the gene *cur_1714* within the known genome sequence of *C. urealyticum* codes for the cell wall channel. The study is the first report of the cell wall channel in the pathogenic *C. urealyticum* and could help in better understanding of passage of hydrophilic solutes across the cell wall and developing new drugs against this opportunistic pathogen causing urinary tract infections.

## Methods

### Bacterial strains and growth conditions

The *C. urealyticum* DSM 7109 strain obtained from Deutsche Sammlung von Mikroorganismen und Zellkulturen was grown in 1000 ml baffled Erlenmeyer flasks containing 250 ml of brain-heart infusion (BHI) medium (Becton) and 250 ml Erlenmeyer flasks containing 25 ml BYT medium [34]. The *Corynebacterium* strains *C. glutamicum* Δ*porH*Δ*porA* [30] and *C. glutamicum* Δ*porH*Δ*porA* expressing *cur_1714* were grown in BHI broth and were stirred on a rotary shaker at 200 rpm and 30 °C. *Escherichia coli* NEB5α (New England Biolabs) and *E. coli* BL21 DH5α (ThermoFisher Scientific) were used for cloning and grown under standard conditions in Luria broth (LB) at 37 °C, shaken at 250 rpm. Agar plates and liquid media were supplemented with 25 μg/ml chloramphenicol, respectively, if required. Porin deficient *BL21 DE3 Omp8 E. coli* strains [30] were used for GST tagging and expressing of *cur_1714* and *cur_1715*, and were grown at 37 °C under standard conditions in Luria broth (LB) while shaking at 250 rpm. Agar plates and liquid media were supplemented with kanamycin (25 μg/ml) or ampicillin (100 μg/ml) respectively, if necessary.

### Cloning of cur_1714 and cur_1715

#### Construction of a C-terminal His_8_-tag

The gene *cur_1714* and its putative ribosome binding site was amplified by PCR from genomic DNA of *C. urealyticum* DSM 7109. The expression vector pXHis, a derivative of pXMJ19 was used for expression [35]. The vector is a shuttle vector which can express proteins in both Gram-positive and Gram-negative bacteria and confers chloramphenicol resistance to the transformed cells. The gene *cur_1714* was amplified by PCR with 1× Fermentas Taq buffer, 0.2 mM dNTPs, 2 mM MgCl_2_, 1 U Taq polymerase (Fermentas) and 0.2 pmol primers; Fwd_1714_XbaI and RP-cur 1714-KpnI pXHis (Table 1). The PCR product was cleaned up from other components by using Qiagen PCR clean up kit, was ligated into the pGEM-T easy vector and transformed in *E. coli* BL21 DH5α competent cells. After confirming the plasmid construction by sequencing with primers M13 reverse and T7 Promotor (Table 1) (GATC Biotech, Cologne, Germany), the *cur_1714* gene and its flanking regions were double-digested from the pGEM-T easy vector using *XbaI* and *KpnI* (Fermentas). The gene *cur_1714* was cloned and ligated into the expression shuttle vector pXMJ19 containing an octahistidine-tag at the C terminal end for efficient purification of the protein. This tag is cleaved later with Factor Xa protease at its recognition site (I-E-G-R).

**Table 1:** Oligonucleotides used in this study for cloning of the genes *cur_1714* and *cur_1715.* The sequences of the primers were derived from the prospective genes *cur_1714* and *cur_1715* and their flanking regions taken from the genome of *C. urealyticum DSM 7109* [13]

Oligonucleotides	Sequence 5’➝ 3’
Fwd_1714_XbaI	GTGTCTAGAGACCTACACTCTAGGAGTTTC
RP pX cur_1714 KpnI	CTGGTACCTTAGAAGCCGAATGCCTG
RP-cur1714-KpnI pXHis	GCTTAAAGGTACCGAAGCCGAATG
RevpXMJ19	CAGACCGCTTCTGCGTTCTG
Fwd Gst 1714 BamHI	CTGAGGATCCGGTAACGCAAC
Rev Gst 1714 EcoRI	GACGAATTCTTAGAAGCCGAATGC
Fwd Gst 1715BamHI	CAGTGGATCCAACGTCGACATG
Rev Gst 1715 EcoRI	CTAGAATTCCTACTTGTTCTCGG
Fwd GST Seq	CACTCCCGTTCTGGATAATG
Rev GST Seq	CACTCCGCTATCGCTACGTGAC
T7 Promotor	TAATACGACTCACTATAGGG
M13 reverse	CAGGAAACAGCTATGAC

Recognition sites for the restriction enzymes are underlined

Fwd_1714_XbaI and RP pX cur_1714 KpnI pairs of primers were used for cloning the *cur_1714* gene in pXMJ19 without His tag under the same conditions. Transformation into *C. glutamicum* Δ*porA*Δ*porH* was performed by electroporation using a slightly modified electro-transformation method [36]. Heterologous expression of the protein was induced by addition of 1 mM IPTG (isopropyl-beta-D-thiogalactopyranoside) to a liquid culture in the mid-exponential growth phase. Besides the C-terminal octa-histidine tag we introduced also an N-terminal GST-tag on Cur_1714 and Cur_1715. For the expression of *C. urealyticum* porins a pGEX-2 T expression vector (Amersham Biosciences, GE Healthcare, and UK) was used. The vector contains *glutathione S-transferase* (*GST)* as a fusion *tag and a specific cleavage site (L-V-P-R) for thrombin at the N-terminus* for efficient cleavage of the tag from the protein. Due to the N-terminal fusion, the first methionine was removed.

### Colony PCR and selection of transformed colonies

Colony PCR of *C. glutamicum* Δ*porA*Δ*porH* with pXMJ19 *cur_1714* and pXHis *cur_1714* was performed using slightly modified standard conditions with the specific primers for the insert to check colonies containing the required gene before sequencing. For this issue, single cell colonies were selected and transferred into a 1.5 ml Eppendorf tube containing 20 μl of distilled water. Then, the culture was given seven times 10 s heat shock with 5 s interval between every heat shock. 1 μl of the sample was used as a template for the colony PCR reaction and the correct transformants were checked by sequencing (GATC Biotech, Cologne, Germany).

### Expression of recombinant his tag proteins

Heterologous expression of the protein was induced by addition of 1 mM IPTG (isopropyl-beta-D-thiogalactopyranoside) to a liquid culture at the mid-exponential growth phase.

### Isolation of recombinant cell wall proteins

Cell wall-associated proteins were isolated by methods described previously in detail [37, 38]. A liquid culture of grown cells was centrifuged (4000 rpm, 15 min, 4 °C in an Eppendorf centrifuge 5810R rotor A-4-81). The cell pellet was washed twice with 10% culture volume (10 mM Tris-HCl, pH 8) before cell wall proteins were extracted either by shaking the cells in detergent solution or in a 1:2 (*v*/v) mixture of the organic solvents chloroform and methanol. For both extraction methods one part cells (0.3 g wet weight bacterial cell pellet) was resuspended in five parts detergent solution (1.5 ml 1% LDAO (lauryldimethylamine-oxide), 10 mM Tris-HCl, pH 8) or organic solvent (1.5 ml chloroform/methanol). After 3 h agitation at room temperature, cells were sedimented in a table top centrifuge (Microcentrifuge 5415R) (10 min, 4 °C, 10,000 rpm) and the pellet was discarded. The detergent supernatant was immediately applied to IMAC purification. The chloroform-methanol mixture had first to be precipitated with 9 times the volume of ice-cold diethyl ether (16 h, − 20 °C) before the obtained pellet was either resolved in detergent solution (1% LDAO, 10 mM Tris, pH 8) or in loading buffer for gel electrophoresis [37].

### IMAC purification

The histidine-tailed *C. urealyticum* protein was purified to homogeneity by utilization of immobilized metal ion affinity chromatography (IMAC). From detergent treated cells 5 ml of the 1% LDAO supernatant were loaded on Ni-NTA spin columns (Qiagen) equilibrated with buffer 1 (20 mM Tris, 50 mM NaCl, 0.4% LDAO, pH 8). After ten washing steps using each 650 μl of buffer 2 (= buffer1 with 10 mM imidazol) bound protein was eluted from the column with 200 μl buffer 3 (= buffer 1 with 300 mM imidazol).

### Expression and isolation of recombinant GST-tagged cur_1714 *and* cur_1715

The plasmids including desired genes were sequenced and transformed into the porin deficient *BL21 DE3 Omp8 E. coli.* Cells were grown at 37 °C in LB medium and induced by 1 mM IPTG. The culture was incubated in media overnight at 28 °C after induction., cells were harvested by centrifugation at 6000×g for 20 min at 4 °C and were resupended in PBS (phosphate buffered saline) (pH 7.4). Subsequently the cells were lysed using a high-pressure-homogenizer (2 × 1500 bar). Unbroken cells were removed by centrifugation, the supernatant was used for purification.

### GST-PorACur fusion protein purification

Purification of GST-tag proteins were performed using glutathione Sepharose 4B medium, (following batch protocol GST Gene Fusion System Handbook, GE Healthcare). Glutathione is a tripeptide (Glu-Cys-Gly) and the specific substrate for glutathione S-transferase (GST). When reduced glutathione (GSH) is immobilized through its sulfhydryl group it can be used to capture GST-tagged proteins via the enzyme-substrate binding reaction. After 5 times washing with buffer A (0.5% Genapol, 100 mM NaCl, 50 mM Tris-HCl, 1 mM CaCl_2_, 2.5 mM DTT, pH 7.4) to remove non-bound sample components, the purified GST-fusion protein was eluted by addition of buffer 1 supplemented with 10 mM glutathione, pH 8. The protein sample was concentrated using Amicon ultra 3 kDa to a concentration of GST-fusion proteins.

### Protease Xa cleavage of C-terminal His_8_ of *C. urealyticum* proteins

Subsequent to IMAC purification the sample contained high imidazol concentrations which strongly inhibit protease Xa (Qiagen) activity. Removal of imidazol was performed by dialyzing the sample over night against cleavage buffer (20 mM Tris, 50 mM NaCl, 1 mM CaCl_2_, 0.4% LDAO, pH 6.5) using a cellulose membrane with a MWCO of 2 kDa (Spectra/Por 6, Carl Roth, Germany). For cleavage of the poly-histidine-tag four units protease Xa (Qiagen) were added to the sample (23 °C, overnight). The enzyme was removed with the factor Xa removal Kit according Qiagen instructions while the cleaved protein was separated from uncleaved PorACur-His_8_ by a second passage through a Ni-NTA filter.

### Thrombin cleavage

In order to remove the GST-tag from purified protein it was incubated for 10 h at room temperature with 100 μl of thrombin agarose resin and 100 μl of 10 X cleavage buffer (500 mM Tris-HCl, pH 8.0, 100 mM CaCl_2_). The pure recombinant protein was purified from GST fusion protein by using preparative SDS-PAGE.

### Protein electrophoresis and immunoblotting of the C-terminal his-tag

Analytical and preparative SDS-PAGE was performed according to Laemmli [39] subsequent to a denaturation step (5 min, 95 °C). Because of the low resolution of this gel system for the detection of low molecular mass proteins, tricine containing gels containing 15% or 16.5% polyacrylamide were used as described by Schägger and von Jagow [40]. After electrophoresis, gels were either stained with Coomassie Brilliant Blue G-250, or by silver stain [41] or electroblotted [42]. In the latter case, proteins were transferred to a 0.1 μm nitrocellulose membrane (Protran, BA79, Whatman). The blotting was performed for 4–5 min in a wet tank blot system (Biorad) with Towbin buffer (25 mM Tris, 192 mM glycine, 20% methanol) at 350 mA current. Unspecific binding sites on the membrane were blocked with 5% skimmed milk in TBS-T buffer (20 mM Tris, 0.01 M NaCl, 0.1% Tween, pH 7.5) before probing with the first 1: 5000 diluted monoclonal Anti-his antibody (Amersham Biosciences, UK). Subsequent to multiple TBS-T washing steps the second peroxidase-conjugated Anti-Mouse antibodies (DAKO, Denmark) were added at the same dilution. According to the manufacturer’s instructions the use of the ECL Western blotting detection system (GE Healthcare, UK) resulted in light emission recorded on autoradiography films (Hyperfilm™MP, GE Healthcare, UK). Dot blot immune detection was carried out identically without prior SDS-PAGE. Exposure times varied between 10 s to 5 min as required by the sample.

### Protein electrophoresis and immunoblotting of the N-terminal GST-tag

Proteins were transferred to a 0.45 μm membrane. Subsequently, the membrane was shaken for 1 h in a solution of anti GST antibody (GE Healthcare, UK) at a dilution of 1:3, 000 and then washed three times with Tris-buffer saline, each time 10 min. Anti-Goat IgG bound to alkaline phosphatase from Sigma at a dilution of 1:30, 000 was used as secondary antibody to detect the bound antibodies. The membrane was washed three times with alkaline phosphatase buffer pH 9.5 (0.1 M Tris, pH 9.5 and 0.1 M NaCl) afterward. Alkaline phosphatase activity was detected by colorimetric reaction. To detect the protein bands recognized by specific antibodies, the membranes were incubated with BCIP (5-bromo-4-chloro-3-indolyl-phosphate)/NBT (nitro blue tetrazolium) (Sigma) as substrate until the bands appeared.

### Black lipid bilayer membranes

The device used for the black lipid bilayer experiments has been described in detail [43]. The basic part is a Teflon cell with two aqueous compartments connected by a circular hole with a small area. The lipid bilayers were formed by painting onto the hole a 1% (*w*/*v*) solution of diphytanoyl phosphatidylcholine (Diph-PC)/n-decane (Avanti Polar Lipids, Alabaster, USA). The aqueous salt solutions were used unbuffered and the temperature during all experiments was kept at 20 °C. Two Ag/AgCl electrodes with salt bridges were inserted in the compartments that were connected in series with a battery-driven voltage source and a highly sensitive current amplifier. Its output signal was monitored with a digital oscilloscope and recorded with a strip chart recorder. The zero-current membrane potential measurements were performed as it has been described earlier [44] by establishing a 5-fold salt gradient across membranes containing about 100 cell wall channels. The potentials were measured using a high impedance electrometer (Keithley 617), and analyzed using the Goldman-Hodgkin-Katz equation [44].

### Molecular dynamics simulations

Initial models of hexamer and octamer channels were constructed using the CCBuilder tool [45] without prior structural information. Both channels were simulated in a POPE bilayer containing a 1 M KCl solution (hexamer system about 66.000 atoms, octamer system about 90.000 atoms) using GROMACS 5.1.2 [46] and the CHARMM36 force field [47, 48]. Long-range electrostatic potentials beyond the cut-off of 12 Å were computed using the particle mesh Ewald algorithm [49]. Lennard-Jones potentials were computed using the 10–12 Å force-switch scheme. The systems were equilibrated for 16 ns following the protocol described in the CHARMM-GUI Membrane Builder [50]. Unbiased production runs of 500 ns each were performed for the two systems in the pressure ensemble. The Nosé-Hoover thermostat [51] was used to maintain a constant temperature of 303.15 K by coupling the protein, lipid and solvent separately to a temperature bath using a coupling constant of 1 ps. Moreover, the pressure was maintained constant at 1 bar by using the Parrinello-Rahman barostat [52] with semi-isotropic scaling with a coupling constant of 5 ps. Covalent bonds containing hydrogen atoms were constrained using the LINCS algorithm [53] to be able to use an integration time step of 2 fs. Furthermore, two simulations of the octamer system were performed in the presence of an applied voltage of 100 mV to determine the ion conductance at 1 M KCl. The membrane potential (V = E/L_z_) was modelled by applying a constant electric field (E) perpendicular to the membrane [54, 55].

## Results

### Cell wall proteins from *C. urealyticum* effect the conductance of lipid bilayer membranes

**Fig. 1:**
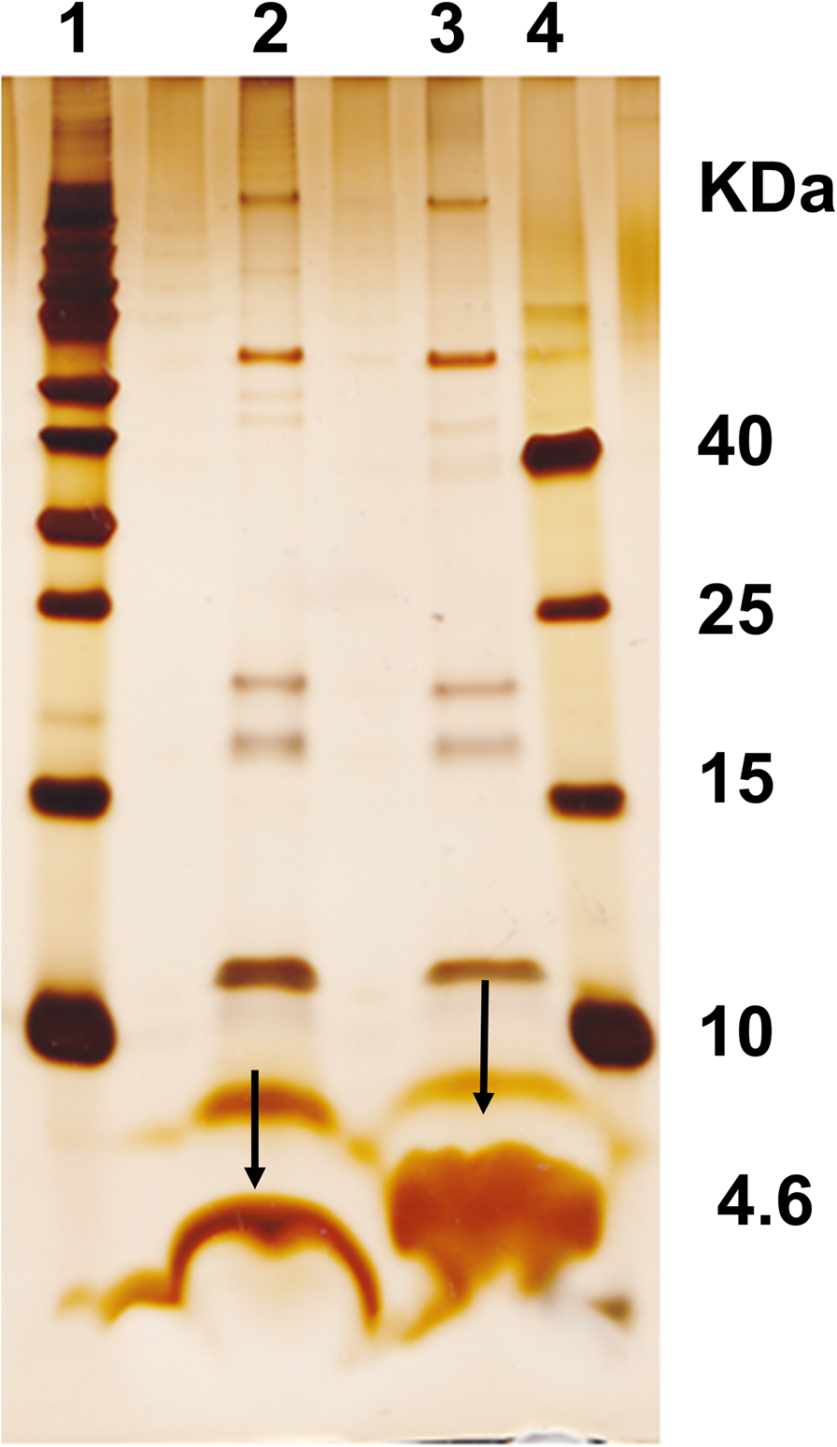
Extracts from whole *C. urealyticum* cells visualized on a 16.5% SDS-PAGE containing tricine. The SDS-PAGE was stained with silver. Lane 1: High molecular mass markers, Lane 2: whole *C. urealyticum* cell wall extract, heated at 95 °C for 10 min Lane 3: whole *C. urealyticum* cell wall extract (not heated) and Lane 4: Low molecular mass protein markers (4.6, 10, 15, 25 and 40 kDa). The arrows indicate the gel location of the putative pore-forming protein

Cells of a *C. urealyticum* culture grown overnight were extracted with 1% LDAO. A few μl of the crude cell wall extract were tested in the lipid bilayer assay for pore-forming activity. Conductance steps with 1.75 nS were observed at 20 mV applied membrane potential in 1 M KCl solution. Furthermore, the conductance increase caused by the detergent extract was a function of time after the addition of the protein to membranes in the black state. Control experiments with LDAO alone at the same concentration as in the experiments with extracts demonstrated that the membrane activity was caused by the presence of the extracts and not by the detergent. This result suggested that the channel-forming activity was an intrinsic property of the detergent extracts of whole *C. urealyticum* cells. The channel-forming proteins were further purified by excising the enriched bands from preparative Tricine-PAGE gel and extracting the gel slice with 1% Genapol X-80. Addition of the extracted 4 kDa band to black lipid bilayer membranes resulted in reconstitution of channels. When regions at different molecular masses were excised from the same Tricine-PAGE gel, the highest channel-forming activity was always combined with the low molecular mass 4 kDa band. However, we also noticed that some minor channel-forming activity was spread across the molecular mass region between about 4 and 40 kDa (Fig. 1) of the tricine-containing SDS-PAGE.

### Single-channel analysis of the pores formed by the *C. urealyticum* cell wall extract

**Fig. 2:**
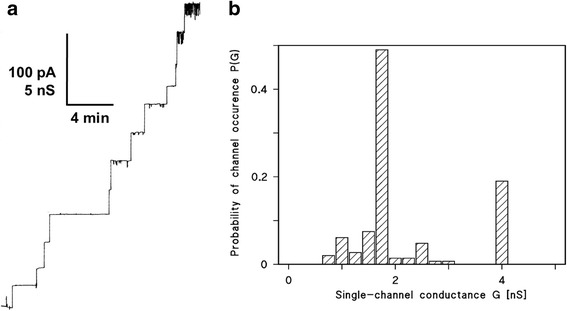
**a** Single-channel recording of a Diph-PC/*n*-decane membrane in the presence of detergent extract of whole *C. urealyticum* cells. The aqueous phase contained 1 M KCl, pH 6 and about 50 ng/ml protein extract. The applied membrane potential was 20 mV; *T* = 20 °C. **b** Histogram of the probability P(G) for the occurrence of a given conductivity unit observed with membranes formed of 1% Diph-PC dissolved in *n*-decane. The histogram was calculated by dividing the number of fluctuations with a given conductance unit by the total number of conductance fluctuations in the presence of detergent extracts of whole *C. urealyticum* cells. Two frequent conductive units were observed for 147 single events taken from 6 individual membranes. The average conductance of the steps corresponding to the left-side maximum was 1.75 nS and that of the right-side maximum was about 4.0 nS. The aqueous phase contained 1 M KCl, pH 6 and about 50 ng/ml protein extract, the applied membrane potential was 20 mV, T = 20 °C

In single channel experiments with lipid bilayers, we observed a considerable increase of the specific membrane conductance in the presence of *C. urealyticum* cell wall extracted with 1% LDAO. The addition of lower concentrations of the cell wall extract to lipid bilayers made of Diph-PC/*n*-decane allowed the resolution of stepwise conductance increases. Figure 2a shows a single channel recording in the presence of the *C. urealyticum* cell wall extract, 5 min after the addition of the protein to a lipid bilayer membrane. Each step indicates the incorporation of one channel forming unit into the membrane. All steps were directed upwards, which demonstrated that the channels were always in the open state and had a long lifetime. Figure 2b shows a histogram of all conductance steps observed in reconstitution experiments with the *C. urealyticum* cell wall extract in 1 M KCl and a voltage of 20 mV. The most frequent value for the single-channel conductance was 1.75 nS, another less frequent conductive unit had a conductance of about 4 nS.

The single-channel conductance of the pore-forming protein from *C. urealyticum* cell wall extract was also studied as a function of different KCl concentrations (Table 2) to get some insight into the biophysical properties of the cell wall porin of *C. urealyticum*. Additional information was obtained from single-channel experiments with salts containing other ions than K^+^ and Cl^−^. The results summarized in Table 2 demonstrated that the channel was only moderately selective. This is concluded from experiments in which KCl was replaced by LiCl or KCH_3_COO. The exchange of the mobile ions K^+^ and Cl^−^ by the less mobile ions Li^+^ and acetate showed that cations and anions have certain permeability through the *C. urealyticum* channel. The permeability of the cations through the channels followed approximately their mobility sequence in the aqueous phase. This means probably that the cell wall porin of *C. urealyticum* forms a wide and water-filled channel that exhibited some selectivity for cations.

**Table 2:** Average single-channel conductance of the pore formed by the detergent extract of *C. urealyticum* in different electrolyte solutions

Electrolyte	Concentration (M)	G (nS)
KCl	0.01	0.04
	0.03	0.1
	0.1	0.2
	0.3	0.6
	1	1.75
	3	5
LiCl	1	1
KCH3COO (pH 7)	1	1.25

The membranes were formed of 1% DiphPC/*n*-decane. The aqueous solutions were unbuffered and had a pH of about 6 if not indicated otherwise. The applied voltage was 20 mV and the temperature 20 °C. The single values represent the means (± SD) of at least 100 single-channel events derived from at least four individual membranes

### Selectivity measurements with pores formed by the *C. urealyticum* cell wall extract

Further information about the channel observed in *C. urealyticum* cell wall extract was obtained from zero-current membrane potential measurements in the presence of salt gradients. Fivefold salt gradients (100 mM versus 500 mM) were established across lipid bilayer membranes in which about 100 to 1000 channels were reconstituted. The results are summarized in Table 3 and suggested indeed that the cell wall pore of *C. urealyticum* is slightly cation selective.

**Table 3:** Zero-current membrane potentials, V_m_, of Diph-PC/*n*-decane membranes in the presence of the pore formed by the detergent extract of *C. urealyticum*, measured for a 5-fold gradient of different salts

Electrolyte	Permeability ratios P_cation_/P_anion_	V_m_ (mV)^a^
KCl	2.4	14 ± 3
LiCl	0.54	−8.9 ± 0.2
KCH_3_COO (pH 7)	7.8	24 ± 2

^a^The potential *V*_*m*_, measured for fivefold gradients of different salts, is defined as the difference between the potential on the dilute side (100 mM) and the potential on the concentrated side (500 mM). The aqueous salt solutions were used unbuffered with a pH of 6, if not indicated otherwise. The temperature was 20 °C. The cation/anion permeability ratio was calculated as the mean of at least 3 individual experiments [44]

### Estimation of the diameter of the cell wall porin of *C. urealyticum*

Experiments with non-electrolytes (NEs) were performed according to an established method to estimate the effective pore diameter of the pore in the cell wall of *C. urealyticum* [56–62]. The 1 M KCl solutions were supplemented for these experiments with 20% (*w*/*v*) NEs with different molecular masses ranging from 62 Da (ethylene glycol) to 2 kDa (PEG 2000) and molecular radii between 0.26 nm and 1.22 nm (Table 4). The results of single-channel conductance measurements with these NEs suggested that in the presence of small NEs with hydrodynamic radii up to 0.94 nm, the single-channel conductance decreased, while PEG 2000 with hydrodynamic radii of 1.22 nm did not enter the *C. urealyticum* channel and showed no effect on its conductance (Table 4). This means that the pore in the cell wall of *C. urealyticum* has an exclusion limit below 2 kDa.

**Table 4:** Estimation of the diameter of the cell wall pore of *C. urealyticum*

Non-electrolytes (Nes)	Mr (g/mol)	r (nm)	G (pS)	*X* (mS cm^− 1^)
None	–	–	1750 ± 250	110.3
Ethylene glycol	62	0.26	800 ± 100	57.2
Glycerol	92	0.31	500 ± 100	49.1
Arabinose	150	0.34	700 ± 100	63.7
Sorbitol	182	0.39	800 ± 100	57.8
Maltose	360	0.50	800 ± 100	73.8
PEG 300	300	0.60	1000 ± 100	45.5
PEG 400	400	0.70	1000 ± 100	46.4
PEG 600	600	0.80	1000 ± 100	54.1
PEG 1000	1000	0.94	1300 ± 100	49.5
PEG 2000	2000	1.22	1700 ± 250	43.6

The aqueous phase contained 1 M KCl and 20% (w/v) of different non-electrolytes. The non-electrolytes were used to determine the diameter of the cell wall pore. The molecular masses and the hydrodynamic radii (r) of the non-electrolytes are given together with the conductance of the channel (G ± SD) in the corresponding solution and the aqueous conductivity. *X* is the aqueous conductivity of the salt solutions with and without the NEs and was taken from ref. [62]. The membrane voltage was 20 mV; *T* = 20 °C

### The cell wall channel of *C. urealyticum* is not voltage dependent

To study of the voltage-dependence of the channel from *C. urealyticum*, 200 ng/ml of the cell wall extracts was added to 1 M KCl solution at the cis-side of a Diph-PC/*n*-decane membrane and the reconstitution of the channels was followed for about 30 min. Then increasing positive and negative voltages were applied to the cis-side of the membrane. The *C. urealyticum* cell wall channel did not show any voltage dependence up to ±100 mV (data not shown).

### Identification of the gene coding for the cell wall pore of C*. urealyticum*

**Fig. 3:**
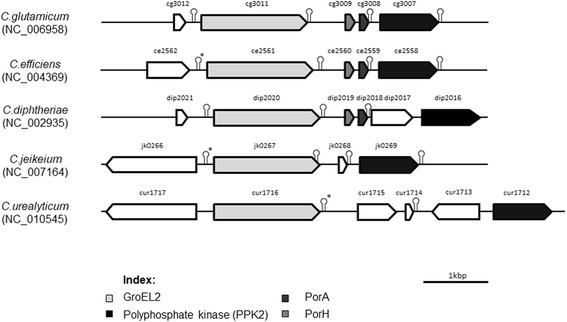
Analysis of the accessible genomes from *C. glutamicum* (ATCC 13032)*, C. efficiens* (YS-314 DNA)*, C. diphtheriae* (NCTC 13129)*, C. jeikeium* (K411) *and C. urealyticum* (DSM 7109)*.* The homologous genes of the chaperonin GroEL2 and a polyphosphate kinase PPK2 enclose a presumed conserved porin domain. The operon covering the genes Cg*porH* and Cg*porA* whose gene products form the main cell wall channel of *C. glutamicum* is assumed to exist in all strains except for *C. jeikeium*. Search within the genome of *C. urealyticum* indicated that this strain does not follow this rule and suggested instead that it contained 3 open reading frames (ORFs) between the genes coding for GroEL2 (*cur_1716*) and PKK2 (*cur_1712*). They may code for cell wall proteins similar to PorA. Possible terminator sequences of mRNA transcripts were predicted with TranstermHP (indicated by hairpins [78] or were identified manually (marked by asterisk))

The extracts from whole *C. urealyticum* cells showed many bands on 16.5% tricine containing SDS-PAGE (Fig. 1). Excision of the low molecular mass bands from preparative SDS-PAGE suggested that they contained the channel-forming protein, but it was impossible to relate one defined single band to the channel-forming activity. Therefore, we looked for an alternative method to identify the channel-forming protein. Previously we could identify PorACj of *C. jeikeium* based on its homology with PorA of *C. glutamicum* and other *Corynebacterium* species [31, 63–65]. Therefore, we performed a similar approach here. A NCBI BLAST-translation tool search [32, 33] using *porA* of *C. glutamicum* in the known genome of *C. urealyticum* DSM 7109 [13] did not lead to a clear indication for a homologous gene. However, search within the genome suggested that it contained three open reading frames (ORFs) between the genes coding for GroEL2 (*cur_1716*) and PKK2 (*cur_1712*) that could code for cell wall proteins similar to PorA (see Fig. 3). Primers were designed to clone *cur_1714* and *cur_1715* separately using DNA of *C. urealyticum* DSM 7109 as a template (Table 1). The PCR-product was cloned into the pGEM-T easy vector and transformed to *E. coli* BL21 DH5α competent cells. The plasmid containing the desired insert was confirmed by sequencing using primers M13 reverse and T7 Promoter (Table 1). The *cur_1714* gene and its flanking regions were double digested from pGEM-T easy vector using *XbaI* and *KpnI* for cloning the gene into the expression vectors. Proteins coded by *cur_1714* and *cur_1715* of *C. urealyticum* DSM 7109 were purified separately by immobilized metal ion affinity chromatography (IMAC) and preparative SDS-PAGE according to Laemmli [39]. Addition of purified GST and His tagged proteins to black lipid bilayer membranes made of PC/*n*-decane did not result in conductance increases. This could mean that the tags had to be removed for pore-forming activity of the two proteins. Histidine residues attached to the C-terminal end of CUR_1714 were cleaved with Factor Xa protease and the GST attached to the N-terminal of CUR_1715 was cleaved with thrombin protease.

Pure CUR_1714 and CUR_1715 proteins where His-tag and GST-tag, respectively, were cleaved did not show any channel-formation in lipid bilayer membranes despite many attempts. This result clearly indicated that the two proteins in this form (i.e. where His-tag and GST-tag were cleaved) did not represent channel-forming components. We also tried to detect channels when both proteins were added together to the membranes. However, also in this case we did not observe any pore-forming activity, which means that these two proteins after cleavage of the affinity tags, neither together nor alone were channel formers.

### Mature CUR_1714 is the channel former in *C. urealyticum* DSM 7109

**Fig. 4:**
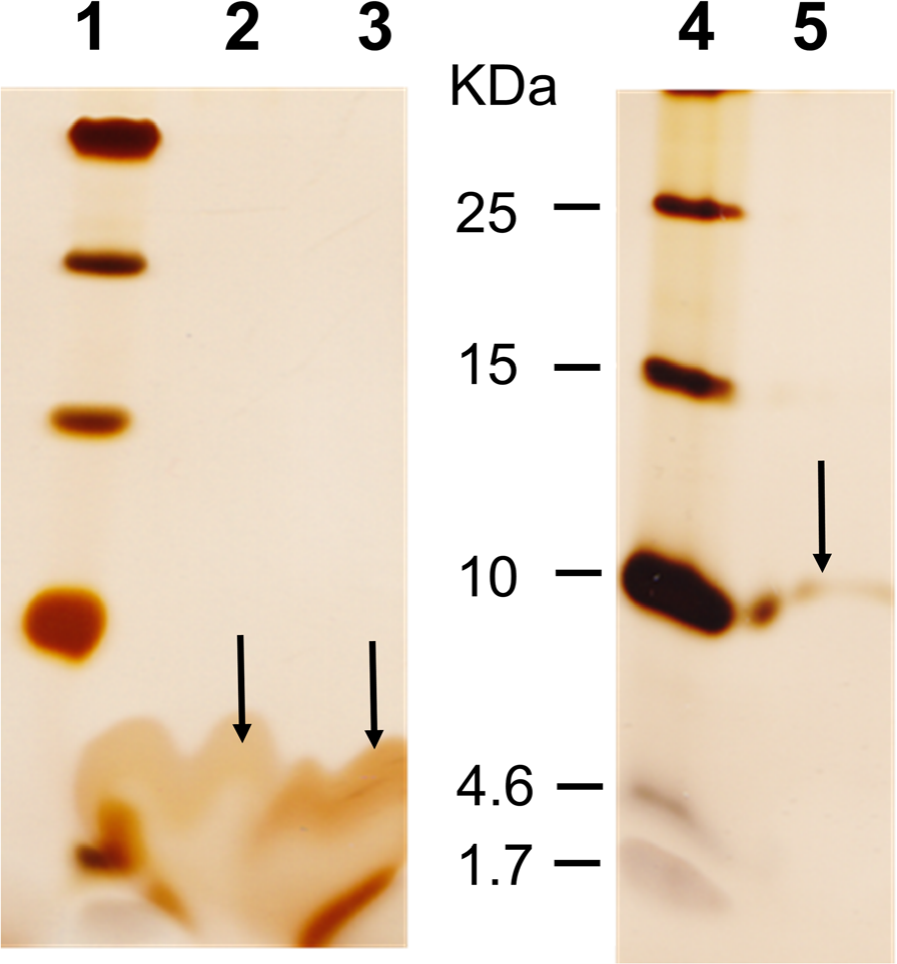
Different preparations of PorACur visualized on a 16.5% SDS-PAGE containing tricine. The SDS-PAGE was stained with silver. Lanes 1 and 4, Low molecular mass protein ladder. Lane 2, Purified PorACur with C-terminal His_8_-tag expressed in *C. glutamicum* ATCC 13032 ∆HA, Lane 3, Purified PorACur from preparative gel without tag expressed in *C. glutamicum* ATCC 13032 ∆HA, Lane 5, Purified CUR_1715 without GST-tag (around 9 KDa) expressed in *E. coli* BL21 DE3 (Omp8). The purified proteins with and without tags were reconstituted into lipid bilayer membranes. Only protein of lane 3 shows channel-formed activity in lipid bilayers. The channel-forming protein, named PorACur (coded by *cur_1714* gene), was identified in the known genome of *C. urealyticum* by its similar chromosomal localization to known *porH* and *porA* genes of other *Corynebacterium* strains (see Fig. 3). The arrows indicate the location of the corresponding proteins

Another problem that had seriously be considered is the possibility that CUR_1714 contained after cleavage with FXa protease still 6 additional amino acids at the C-terminal end that could interfere with channel formation. Four amino acids (IEGR) are leftovers from FXa protease cleavage because they are the recognition site (I-E-G-R) for FXa to facilitate removal of the fusion tags after arginine (R) residue. Additional two amino acids (GT) are the leftovers from *KpnI* restriction enzyme which cannot be avoided due to the cloning strategy. Therefore, we cloned *cur_1714* into the pXMJ19 using Fwd_1714_XbaI and RP pX cur_1714 KpnI primers. The vector contained a cloning site with *XbaI* and *KpnI* restriction sites. After control of the plasmid by sequencing with primers M13 reverse and T7 Promotor (Table 1), *cur_1714* gene and its flanking regions were double-digested from pGEM-T easy vector using *XbaI* and *KpnI*. The gene *cur_1714* was cloned and ligated into the shuttle vector pXMJ19. Next step was the expression of the protein without any tag in *C. glutamicum* Δ*porA*Δ*porH* pXmj19 lacking the major cell wall channel proteins. The transformed cells were grown at 30 °C in BHI medium to an OD_600_ of around 0.6–0.8. Induction of CUR_1714 expression by adding 1 mM IPTG to the growth medium and additional overnight growth. The protein was successfully expressed and purified without any other extra amino acid. For purification analytical and preparative SDS-PAGE was performed [31, 66]. Because of the low resolution of this gel system for observation of low molecular mass proteins, tricine containing gels were used as described previously [40]. Protein samples were size separated with 15% or 16.5% polyacrylamide gels containing Tris-Tricine. After gel size separation, a protein band of ~ 4 kDa was cut out and was homogenized in 1% Genapol and 10 mM Tris-HCl, pH 8 for 20 h at 12 °C under agitation (Fig. 4). Pore-forming activity was present in the resulting supernatant similar to that shown in Fig. 2.

**Fig. 5:**
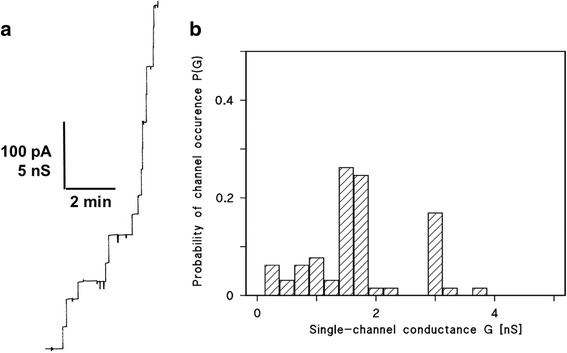
Study of pore-formation in the presence of pure PorACur of *C. urealyticum*. **a** Single-channel recording of a Diph-PC/*n*-decane membrane in the presence of 10 ng/ml pure PorACur. The aqueous phase contained 1 M KCl and the applied membrane potential was 20 mV; T = 20 °C. **b** Histogram of the probability P(G) for the occurrence of a given conductivity unit observed with membranes formed of 1% Diph-PC dissolved in *n*-decane. The histogram was calculated by dividing the number of fluctuations with a given conductance step by the total number of conductance fluctuations in the presence of pure PorACur. Two frequent conductive units were observed for single events taken from different individual membranes. The average conductance of the steps corresponding to the left-side maximum was 1.75 ± 0.25 nS and that of the right-side maximum was 3 nS (65 current steps in total). The aqueous phase contained 1 M KCl, and10 ng/ml protein extract, the applied membrane potential was 20 mV, T = 20 °C

In addition, control experiments with LDAO and 1% Genapol alone at the same concentration as in the experiments with extracts demonstrated that the membrane activity was caused by the presence of the 4 kDa protein extracts and not by the detergent. In reconstitution experiments, we observed a considerable increase of the specific conductance. Single-channel analysis of the purified cell wall porin, named here PorACur (PorA of *C. urealyticum*) showed a conductance of 1.5 ± 0.25 and 3 nS in 1 M KCl (Fig. 5). These channels were identical to those observed after addition of the *C. urealyticum* crude extracts to lipid bilayer membranes. This result suggested that PorACur (CUR_1714) and not CUR_1715 or CUR_1713 represented the cell wall channel of *C. urealyticum*.

### Single-channel analysis of PorACur of C. *urealyticum*

The PorACur protein was successfully expressed and further purified by excising the enriched band from preparative Tricine-PAGE gel and extracting it with 1% Genapol X-80 from the gel slice. Addition of the extracted 4 kDa band to black lipid bilayer membranes resulted in very slow reconstitution of channels. In lipid bilayer single channel experiments, we observed two frequent conductive units of the specific membrane conductance in the presence of pure PorACur of *C. urealyticum* (see Fig. 5). The average conductance of the steps corresponding to the left-side maximum was 1.75 ± 0.25 nS in 1 M KCl and that of the right-side maximum was 3 nS. The single-channel conductance of pure PorACur was also studied as a function of different KCl concentrations. The results are summarized in Table 5 and demonstrate that the single-channel conductance was approximately a linear function of the aqueous salt concentration.

**Table 5:** Average single-channel conductance of PorACur of *C. urealyticum* in different electrolyte solutions

Electrolyte	Concentration (M)	G (pS)
KCl	0.1	300 ± 100
	0.3	600 ± 100
	1	1500 ± 250
	3	5500 ± 500
KCH_3_COO (pH 7)	1	500 ± 100

The membranes were formed by Diph-PC/*n*-decane. The single-channel conductance was measured at 20 mV applied voltage and T = 20 °C. The average single-channel conductance, G (± SD), was calculated from at least 100 single events from up to five individual membranes

### Selectivity measurements with PorACur

Further information on PorACur of *C. urealyticum* was obtained from zero-current membrane potential measurements in the presence of salt gradients. Fivefold salt gradients (100 mM versus 500 mM) were established across lipid bilayer membranes in which about 100 to 1000 channels were reconstituted. Further single-channel analysis indicated that the cell wall channel is wide and water-filled because it is only slightly selective for cations over anions. Its conductance followed the mobility sequence of cations and anions in the aqueous phase. Information on the size and selectivity of PorACur are summarized in Tables 5 and 6.

**Table 6:** Zero-current membrane potentials, V_m_, of Diph-PC/*n*-decane membranes in the presence of the channel-forming protein of *C. urealyticum*, PorACur, measured for a 5-fold gradient of different salts

Electrolyte	Permeability ratios P_cation_/P_anion_	V_m_ (mV)^a^
KCl	2.5	12 ± 2
LiCl	0.57	−8.2 ± 0.2
KCH_3_COO (pH 7)	6.9	23. ± 2

^a^The potential V_m_, measured for a fivefold gradient of different salts, is defined as the difference between the potential on the dilute side minus the potential on the concentrated side. The aqueous salt solutions were buffered with 10 mM Tris-HCl (pH 7), and the temperature was 20 °C. The cation/anion permeability ratio was calculated from at least 3 individual experiments [44]

### Comparison of PorACur with PorACj of *C. jeikeim* K411

**Fig. 6:**
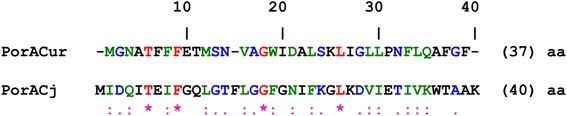
Alignment of sequences of the cell wall protein PorACj of *C. jeikeium* K411 with that of *C. urealyticum* DSM 7109, PorACur, using Clustal W. The alignment was performed using Pole Bioinformatique Lyonnaise Network Protein Sequence Analysis (http://npsa-pbil.ibcp.fr). Amino acids identical in both proteins are highlighted in red (*), strongly similar amino acids (:) are given in green and weakly similar ones (.) in blue

In the following we present a comparison of the sequences of the major cell wall proteins PorACj of *C. jeikeium* K411 with that of PorACur of *C. urealyticum* DSM 7109, because both cell wall pores were obviously formed by a single polypeptide. The multialignment was performed using Pole Bioinformatique Lyonnaise Network Protein Sequence Analysis (http://npsa-pbil.ibcp.fr)*.* Interestingly, we observed some homology between PorACur (coded by *cur_1714* gene) and PorACj as shown in Fig. 6. There exist four amino acids identical (*; 10%) and thirteen amino acids strongly similar (:; 32.5%) in the primary sequence of PorACur (37 amino acids) as compared to PorACj of *C. jeikeium* (40 amino acids)*.*

### Sequence analysis and secondary structure predictions

**Fig. 7:**
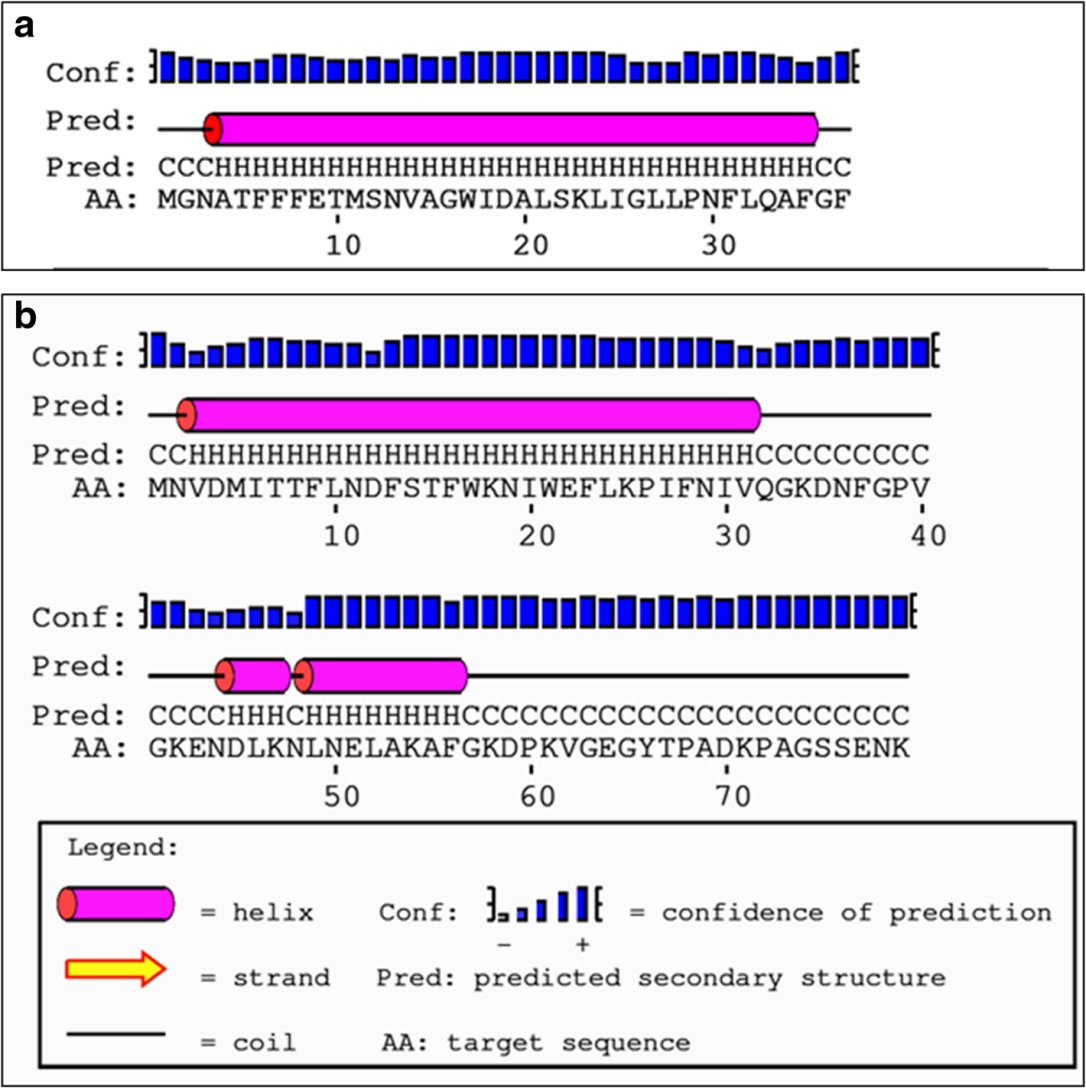
Secondary structure predictions for the putative pore-forming proteins of *C. urealyticum.* (**a**) Prediction for hypothetical protein CUR_1714 and (**b**) Prediction for hypothetical protein CUR_1715. The secondary structure predictions were performed using (http://npsa-pbil.ibcp.fr/cgi-bin/npsa_automat.pl?page=/NPSA/npsa_seccons.html) and suggested that both proteins contained a high propensity forming α-helices

For structural classification and spatial arrangements of secondary structures of potential *C. urealyticum* porins (hypothetical protein CUR_1714 and hypothetical protein CUR_1715) sequences were submitted to different servers such as PSIPRED (http://bioinf.cs.ucl.ac.uk/psipred), MINNOU (http://minnou.cchmc.org/), PredictProtein (http://www.predictprotein.org/), TMRPres2D (http://bioinformatics.biol.uoa.gr/TMRPres2D/) and PORTER (http://distill.ucd.ie/porter/) for predictions of its secondary structure. All secondary structure predictions suggested that these two proteins contained alpha-helices (Fig. 7).

### Putative structure of the channel formed by PorACur

**Fig. 8:**
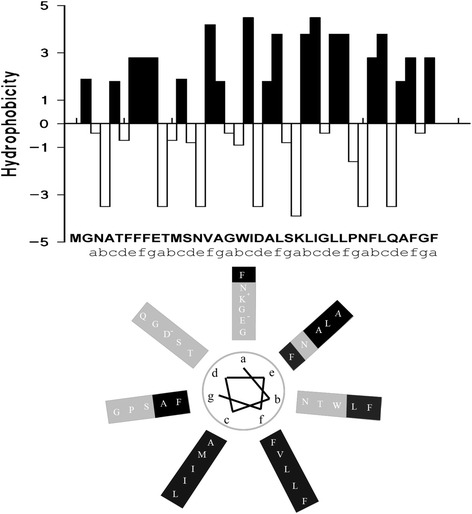
Analysis of the secondary structure of PorACur. **a** The panel shows the hydrophobicity indices of the individual amino acids of PorACur according to [79]. **b** The secondary structure of PorACur was predicted using a consensus method at the Pole Bioinformatique Lyonnaise network (http://npsa-pbil.ibcp.fr/cgi-bin/npsa_automat.pl?page=/NPSA/npsa_seccons.html) to form α-helices. Amino acid residues were arranged on the basis of heptameric repeats (a-g, rotation of 100 degrees per residue starting from a, in the clockwise direction) showing distinct separation in a hydrophobic domain that could be surrounded by lipid molecules (black) while the hydrophilic domain (grey) is suggested to represent the α-helices orientated to the water-filled lumen of the putative oligomeric PorACur channel

Secondary structure predictions suggested that PorACur contained a heptameric repeat motive (abcdefg, Fig. 8) indicating the existence of α-helical structures with hydrophobic and hydrophilic residues localized on different sides of the helices. Figure 8 shows also the possible arrangement of the amino acids within an α-helix PorACur. This means that this protein could form amphipathic helices similar to the possible secondary structure in the monomeric PorH and PorA proteins [28, 29, 64] and also PorACj of *C. jeikeium* [31].

**Fig. 9:**
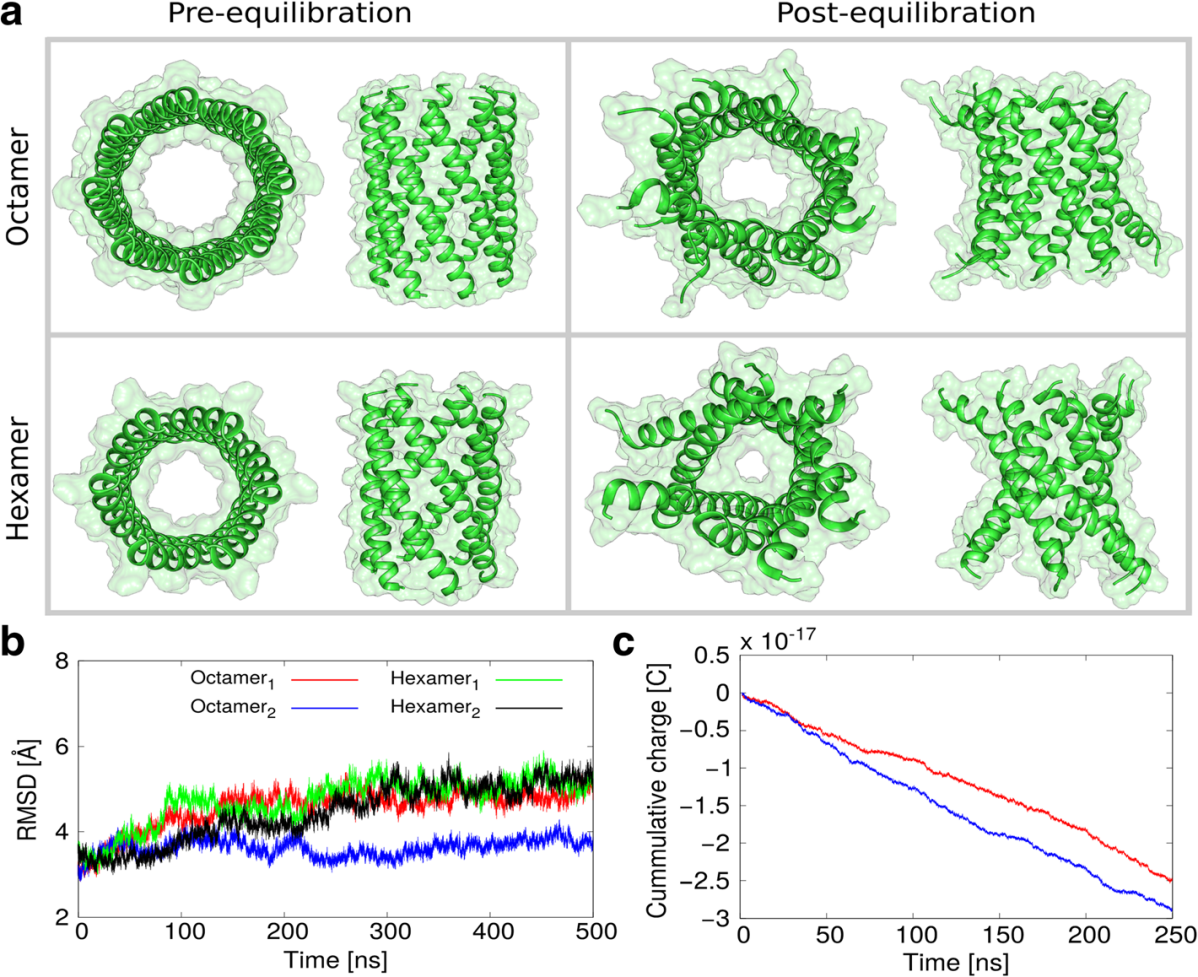
**a** Models of octameric and hexameric channels of PorACur of *C. urealyticum* shown before and after unbiased MD simulations. **b** RMSD profiles of the unbiased trajectories with their respective starting structures as a function of time. **c** Cumulative charge moving through the octamer model at 100 mV and 1 M KCl solution

The PorACur monomer is orientated with the hydrophobic residues (Fig. 8 (f c g)) to the lipid phase, while the two negative residues glutamate and aspartate (E9, D19) together with the positive lysine (K23) are oriented to the channel lumen (a d). Therefore, the negatively charged residues must take a dominant position in the determined cation selectivity. The number of PorACur monomers in the homooligomeric channel is not known yet and needs further experimental and structural information on the channel. Five monomers are suggested to form the PorB channel of *C. glutamicum* [67]. Thus, it is very likely that the PorACur channel is formed by six to eight monomers as it is shown in Fig. 9.

### MD simulation results of PorACur

Initial models of hexameric and octameric channels were constructed as shown in Fig. 9a. Subsequently, unbiased MD simulations were performed on these oligomeric initial models to check their structural and dynamic behavior. Root mean square deviation profiles (Fig. 9b) indicate that the both models deviate by 3–5 Å from starting structure and reach stable conformations after about 250 ns. The octameric arrangement clearly forms a wider lumen than the hexameric channel. The kinking of the α-helices during equilibration is caused by the proline residue present which is known to induce this kind of kinks in α-helices. Furthermore, to determine which of the proposed structures of the channel is more likely, applied-field simulations were performed to compare with experimental electrophysiological data. Applied simulations were performed on the octameric channel bathed in 1 M KCl solution at 100 mV. The calculated average conductance of the octamer model is 1.04 ± 0.14 nS which is in reasonable agreement with the experimental values though lower. Due to the channel diameter, it is obvious that the hexameric model would result in even lower conductance values and thus we refrained from performing the respective simulations. Actually, at this point we cannot rule out that a decameric model would result in conductance values even closer to the experimental findings.

## Discussion

### C. Urealyticum contains a cell wall channel

Treatment of whole cells from the CNM-group of gram-positive bacteria with different detergents provides an efficient and simple way to isolate pore-forming proteins from the cell wall [37, 38]. The same method was also applied to *C. urealyticum* cultures. Lipid bilayer experiments in the presence of the LDAO-extracts demonstrated the presence of a pore-forming protein that formed pores with a single-channel conductance of 1.75 nS in a 1 M KCl solution. This result indicated that the cell wall of *C. urealyticum* contained a pore similar to those of the cell wall of all members of the mycolata studied to date. The pore-forming protein responsible for the formation of the pores had obviously a very small molecular mass because low molecular mass bands in the range of 3 kDa excised from preparative SDS-PAGE showed highest pore-forming activity.

### The cell wall channel of *C. urealyticum* is wide and water-filled

We studied the properties of the cell wall pore of *C. urealyticum*, termed PorACur (see below) in some detail by measuring its single channel conductance and its selectivity. Single channel data and selectivity measurements suggest that the cell wall channel is slightly cation-selective for KCl (with about equal aqueous mobility for K^+^ and Cl^−^) and higher cation-selective for potassium acetate. In the case of LiCl, where the cation has approximately half of the aqueous mobility of Cl^−^, the pore appeared to be slightly anion-selective. This result suggested that the different ions move inside the channel in a similar way as in the aqueous phase because the pore becomes anion-selective in the case of the less mobile Li^+^ ions. This means that the pore appears to be wide and water-filled.

But what is the exact diameter of the cell wall channel PorACur of *C. urealyticum*? This is an interesting question important also for the development of new antimicrobial compounds precisely targeting essential intracellular components. To get some insight in the effective size of the pores formed by PorACur we performed single-channel conductance experiments with non-electrolytes (NEs) according to established methods to measure the exclusion limit of the PorACur channel [56–62]. The results of the measurements with NEs with different molecular masses suggested (see Table 4) that not all NEs were able to enter the channel. From the NEs listed in Table 4 only ethylene glycol (62 Da) to PEG 600 were obviously able to enter the PorACur channel to reduce its conductance. This means that the pore allows the free passage of molecules with a diameter of 1.6 nm and below because the conductance in the presence of these NEs decreased approximately proportional to that of the bulk aqueous conductivity *X* (see Table 4). The conductance in the presence of PEG 2000 did not change in comparison with that of 1 M KCl alone suggesting that this NE with hydrodynamic radius around 1.22 nm did not enter the PorACur channel. The single-channel conductance also decreased for PEG 1000 (hydrodynamic radius 0.94 nm) somewhat, but interestingly much less than that of the specific conductivity *X* of the aqueous bulk solution (see Table 4). This result indicated that there is break of entry of NEs into the PorACur channel around PEG 1000 indicating that the radius of the channel is close to that of PEG 1000, which means that the diameter of the PorACur pore formed is approximately 1.8 nm. Similar diameters have been found for the different cell wall channels of *Corynebacterium* species, such as *C. glutamicum*, *C. efficiens*, *C. diphtheriae* and *C. jeikeium* [29–31, 37, 65]. However, such a diameter is by far larger than diameters of channels in nerve and muscle tissues. This has to do with the function of the different channels. Bacteria live in relatively dilute environment indicating that the channels cannot be too small for the efficient scavenging of nutrients.

The diameter of the pore formed by PorACur is definitely large enough to account for the high susceptibility of *C. urealyticum* for a variety of antibiotics. Fluoroquinolones, ketolides, rifampicin, and tetracyclines are presumably small enough to pass through the cell wall channel of *C. urealyticum*, which means that they can enter the cells [16, 18]. Beta-lactam antibiotics, macrolides and aminoglycosides are more bulky. For these antibiotics the PorACur pore could offer some barrier for their diffusion into the cells causing some sort of intrinsic resistance against them [20, 21, 25].

### The C. Urealyticum cell wall channel is formed dependent only on Cur_1714

Earlier studies into the cell wall channels of *Corynebacterium* species demonstrated the co-existence of two pore-forming polypeptides PorA and PorH in the cell wall of *C. glutamicum*, *C. diphtheriae*, *C. callunae* and *C. efficiens* [28–30]. In addition, results from RT-PCR experiments suggested co-transcription of the corresponding genes *cgporA* and *cgporH of C. glutamicum*, which constitute an operon**.** The important question of this study was whether the cell wall channel of *C. urealyticum* is also formed by two polypeptides like in the *Corynebacterium* species mentioned above or whether the situation is like in *C. jeikeium* where the cell wall channel is formed by a single polypeptide, PorACj [31]. The analysis of the corresponding gene region in the genome of *C. urealyticum* demonstrated that 3 open reading frames (*cur_1713, cur_1714 and cur_1715*) were located between the genes coding for GroEL2 (cur_1716) and PKK2 (cur_1712), where in the genomes of other *Corynebacterium* species only one or two open reading frames were found (see Fig. 3). One gene (*cur_1713*) is transcribed in the opposite direction to *cur_1714* and *cur_1715* and its gene product (molecular mass 25.9 kDa) does not show any homology with that of pore formers. This suggested that this gene does not code for the cell wall channel observed in whole cell extracts. The molecular mass of proteins encoded by *cur_1714* and *cur_1715* are 4.1 and 8.9 kDa respectively. Both genes were separately expressed in the *C. glutamicum* Δ*porH*Δ*porA* mutant strain. The purified proteins were studied in experiments using lipid bilayer membranes. Only Cur_1714 alone, termed PorACur, was able to form ion-permeable channels in these experiments when the protein did not contain N- or C-terminal extensions such as the His_8_-tag or the GST-tag. A comparison of the lipid bilayer data obtained with whole cell extracts and purified PorACur demonstrated that PorACur is identical to the channel-forming protein observed in detergent extracts of whole cells. Cur_1715 did not show any pore-forming activity in lipid bilayer membranes despite many attempts suggesting that it is not a channel-forming component.

### Putative structure of the channel formed by PorACur

Our results demonstrate that PorACur with a molecular mass of 4.1 kDa (37 amino acids) is the channel-former in the cell wall of *C. urealyticum*. Its length is even smaller than PorACj of *C. jeikeium* that has a length of 40 amino acids. PorACur is definitely the smallest polypeptide that forms stable and voltage-independent pores in membranes. All the other low molecular mass pore-forming polypeptides, such as melittin, alamethicin and the defensins form pores that are either voltage-dependent or need often to be initiated by higher voltages [68–70]. The membrane pores formed by all these molecules are oligomers. This most likely also the case for PorACur because it is definitely too small to form a pore on its own. The single PorACur molecule forms obviously an alpha-helical structure with hydrophobic and hydrophilic residues localized on different sides of the helix as suggested by secondary structural predictions (see Figs. 7 and 8). Thus, it is very likely that the membrane pore is formed by several amphipathic PorACur molecules arranged a barrel stave model with an inner diameter of about 1.8 nm. The number of monomers needed for such a structure is an open question but this could influence channel conductance. We consider the possibility of an uneven number of monomers of PorACur in a channel as rather unlikely because the subunits of the PorA/H channel in the cell wall of *C. glutamicum* is presumably a PorA/PorH dimer [30]. In our previous study of the pore formed by PorACj of *C. jeikeium* we made similar considerations and suggested that the pore is very likely formed by an octamer [31].

In this study, we performed applied-field MD simulations of 250 ns length to compare the theoretical conductance of the PorACur membrane pore with the electrophysiological data from single-channel measurements. The results suggested that the octameric pore is definitely more likely because the inner diameter of the hexameric pore is too small to account for the conductance of the cell wall channel of *C. urealyticum*. The conductance of the octameric pore shows reasonable agreement with the experimental values. However, it should be carefully noted that the octamer model is a predicted structure without any prior structural information.

### Comparison of the cell wall channel properties of *C. urealyticum* and other *Corynebacterium* species

The cell envelopes of members of the genus *Corynebacterium* are similar in their composition and organization [71, 72]. Their mycolic acids are relatively short in comparison to those of bacteria of related genera. The known cell wall pores of *Corynebacterium* species share common properties. In particular, they are formed by homo- or heterooligomers of small polypeptides of less than 60 amino acids in contrast to the much bigger MspA homologs of *Mycobacteria*, *Nocardia* and *Tsukamurella* species [73–76]. Similarly, they all have a high propensity to form alpha-helical structures to form wide and water-filled pores [27, 28, 30, 31, 37]. Common to PorA and PorH polypeptides is also that the polypeptides contain no presequences suggesting that they are translated across the cytoplasmic membrane and integrated into the cell wall by a still unknown export system. Many of the cell wall channels of *Corynebacterium* species are formed by PorA/PorH subunits, i.e. they are heterooligomeric. However, two examples now exist, where the cell wall pores are homooligomeric, PorACj of *C. jeikeium* [31] and PorACur of *C. urealyticum*, studied here. Analysis of the primary sequences of the different subunits of the cell wall channels provides a clear phylogenetic relationship between them. They form a superfamily of proteins analogous to the MspA cell wall channel family of *Mycobacterium smegmatis* and related species [73–76] or PorACoram of *Corynebacterium amycolatum* that belongs to the DUF 3068 (Domains of Unknown Function 3068) superfamily of proteins, mainly found in bacteria from the family *Corynebacteriaceae* [77].

**Fig. 10:**
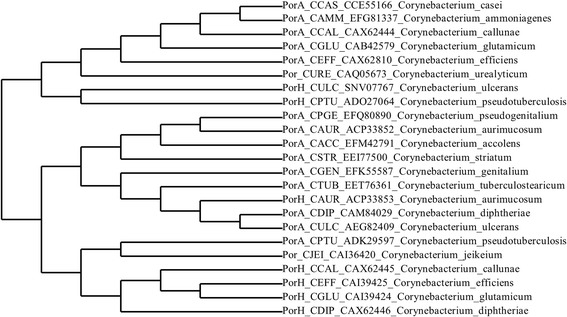
Cladogram representing the phylogenetic relationships of porin proteins of *Corynebacterium* species. The tree was generated with the phylogeny.fr tool [80] using protein sequences downloaded from the NCBI protein database with the indicated identifiers. The multiple sequence alignment was calculated with MUSCLE using the custom mode with a maximum number of 16 iterations. The phylogenetic analysis was performed with PhyML and the approximate likelihood-ratio test for branch support. The substitution model was used in default settings. The tree was rendered with TreeDyn and default settings

The subunit of a monomeric cell wall channel of *Corynebacterium* species, such as PorACj of *C. jeikeium* or PorACur studied here could be the ancestor of the PorA and PorH subunits of the heterooligomeric cell wall channels. In these cases, either PorA-homologs or PorH-homologs could have evolved by gene duplication of an ancestral gene. A phylogenetic tree of the PorA/H family of subunits of cell wall channels of *Corynebacterium* species together with PorACur and PorACj from *C. urealyticum* and *C. jeikeium* is shown in Fig. 10. The tree demonstrates that these two subunits are somewhat distantly related to another although they are both short polypeptides with a high propensity to form alpha-helical structures (see also Fig. 7). Both polypeptides are related to subunits of the corresponding cell wall channels that are obviously formed by PorA and PorH. This could mean that gene duplication occurred several times. It could also mean that gene duplication occurred after a long time when the single cell wall channel subunit existed for already a longer period of time and spread to new *Corynebacterium* species. The interesting point in this relationship is that the PorA family of proteins is more closely related to PorACur and PorACj than the PorH family.

## Conclusions

This paper describes the identification, purification and characterization of the cell wall channel of the pathogenic actinomycete *Corynebacterium urealyticum*. The pore is encoded by the gene *cur_1714* that codes only for the mature protein and not for a protein precursor indicating a not yet identified mechanism for the translocation of PorACur across the cytoplasmic membrane. The channel is formed by small polypeptide subunits that comprise only 37 amino acids, which means that PorACur is the smallest subunit of a cell wall channel from *Corynebacterium* species that forms a stable pore. Secondary structure predictions suggest that the PorACur polypeptide forms an alpha-helical structure. The pore is slightly cation selective and voltage-independent for voltages up to ±100 mV. Single-channel experiments with different salts and non-electrolytes indicate that it is wide and water-filled. Molecular dynamics simulations suggest that 8 to 10 PorACur monomers form the homooligomeric cell wall channel.

## Abbreviations

Diph-PC: Diphytanoyl phosphatidylcholine
GroEL2: 60 kDa chaperonin 2
IMAC: Metal ion affinity chromatography
IPTG: Isopropyl-beta-D-thiogalactopyranoside
PKK2: Polyphosphate kinase
SDS-PAGE: Sodium dodecyl sulfate polyacrylamide gel electrophoresis
